# Numerical Modeling of a Reactive Liquid–Solid Phase Transformation in Molten Steel

**DOI:** 10.3390/ma19143109

**Published:** 2026-07-20

**Authors:** Guanhui Lu, Zeyu Cao, Pengyang Zhao

**Affiliations:** Department of Engineering Mechanics, School of Ocean and Civil Engineering, Shanghai Jiao Tong University, 800 Dongchuan Road, Shanghai 200240, China; jonas_lgh@sjtu.edu.cn (G.L.); yunxuan@sjtu.edu.cn (Z.C.)

**Keywords:** reactive liquid–solid phase transformation, phase-field method, nonmetallic inclusions, molten steel, dendritic growth

## Abstract

A numerical framework is developed to simulate reactive liquid–solid phase transformations in molten steel by coupling chemical reactions, phase-field evolution, and solute diffusion, together with thermodynamic data for oxide formation. The growth of spherical inclusions is governed by the coupling effects of reaction kinetics, thermodynamic driving force, and interfacial properties, exhibiting a non-monotonic temperature dependence. Simulations of the co-evolution of Al_2_O_3_ and SiO_2_ inclusions further show that the morphology of duplex inclusions is essentially controlled by the balance between oxide-solute supply and transformation kinetics. The introduction of interfacial energy anisotropy reproduces dendritic morphologies, revealing that oxide-solute supply, interfacial anisotropy, and interfacial instability together control directional growth and branching behavior. The proposed framework provides a general approach for modeling reactive multiphase transformations.

## 1. Introduction

Precipitation phenomena driven by chemical reactions are widely encountered in materials processing and metallurgical systems, where the interplay between chemical reactions, solute transport, and phase transformations governs the evolution of microstructures. A representative example arises in molten steel refining, where dissolved elements such as aluminum and oxygen react to form non-metallic inclusions [1]. The size, content, morphology, and distribution of these inclusions critically determine the mechanical performance and service reliability of steel products [2,3], defining so-called *steel cleanliness*.

In aluminum-killed steels, alumina-based inclusions are particularly prevalent, whose formation and evolution play a decisive role in determining final product quality. Owing to their high hardness and melting point, Al_2_O_3_ inclusions exhibit limited deformability during thermomechanical processing, which can significantly deteriorate fatigue resistance, toughness, and ductility by acting as initiation sites for fatigue cracks [4,5,6,7]. Their tendency to agglomerate and sinter during continuous casting poses additional challenges to process stability, such as nozzle clogging [8,9]. Despite extensive studies aimed at mitigating these issues through multi-stage deoxidation and refining strategies, the mechanisms underlying the formation and growth of alumina-based inclusions are still unclear. A common simplification in existing theoretical analyses, particularly those based on classical nucleation theory (CNT), is the treatment of precipitation as a local equilibrium process using constant interfacial energy values. This approach inherently neglects the size-dependent variation in the surface energy potential and, more critically, overlooks the local deviations from equilibrium induced by unavoidable compositional and thermal gradients in a liquid steel environment [10]. As a result, it remains a fundamental challenge to establish a *unified* theoretical framework that is capable of describing simultaneously the coupled dynamics of chemical reactions, solute transport, and phase transformation during precipitation. Such coupled processes have also been widely reported experimentally in high-energy-density materials [11,12], thermophysical systems [13,14], soft matter [15,16], and energy-related applications [17,18], demonstrating the generality of reactive phase transformation as a nonequilibrium phenomenon.

Accurately describing this reactive phase transformation therefore represents a central problem in modeling inclusion evolution. Early theoretical studies demonstrated that chemical systems far from equilibrium can exhibit transition behaviors analogous to equilibrium phase transformations, establishing a theoretical basis for reaction-driven phase transitions [19,20]. Subsequent investigations further revealed the close relationship between chemical reactions and structural transformations near phase transitions, highlighting the role of thermodynamic driving forces and microscopic dynamics [21]. At the continuum scale, thermodynamically consistent frameworks have been developed to unify chemical reactions with phase transformation and transport processes [22,23]. In addition, some efforts have been made to incorporate chemical reaction contributions into nucleation modeling within the framework of classical nucleation theory [24].

To study the complex coupling between chemical reactions and phase transformations in the previous examples, numerical simulations are usually carried out using multi-physics modeling frameworks. Zhang et al. [25] developed a coupled phase transformation–chemical reaction model to investigate the safety behavior of liquid storage tanks under thermal radiation, revealing the synergistic evolution of phase transitions and gas-phase reactions. For energetic materials, Williams et al. [26] established a chemo-thermo-mechanically coupled continuum model to simulate the β→δ phase transformation of energetic materials and its interaction with chemical reactions under thermal loading. Similar multi-physics coupling has also been reported in geothermal engineering systems, where thermo-hydro-chemical models incorporating silica dissolution and precipitation reactions have been used to analyze fracture permeability evolution and heat production performance [27,28].

Among various numerical approaches, the phase-field method provides a thermodynamically consistent continuum framework for describing the nucleation, growth, and morphological evolution of microstructures through free-energy-based formulations, typically implemented via the coupled Cahn–Hilliard and Allen–Cahn equations [29,30]. Owing to its capability to capture interfacial dynamics and solute transport without explicit interface tracking, the phase-field method has become an important tool for investigating reaction-driven microstructural evolution. The phase-field framework has been increasingly employed to study reactive phase transformation systems. Xu et al. [31,32] investigated diffusion-controlled dendritic growth and chemically driven precipitation–dissolution processes using phase-field-based formulations. Fang et al. [33] developed a chemo-mechanically coupled phase-field model to analyze sulfate attack in concrete by coupling diffusion, precipitation, and mechanical damage. In ferrous metallurgy, phase-field methods have also been used to study multiphase flow and inclusion behavior. For example, Zhu et al. [34] simulated steel–slag–gas three-phase flow by coupling the Cahn–Hilliard equation with the Navier–Stokes equations, while Xuan et al. [35] and Liu et al. [36] further explored the dynamic interactions and motion of inclusions in steel–slag systems through phase-field-based multi-physics models.

Despite these theoretical and modeling works, systematic studies focusing on the kinetic characteristics of precipitation in such reactive systems remain relatively limited. Recently, Cao et al. [37] proposed a novel phase-field modeling framework in which chemical reactions are represented through elementary reaction steps and a transition-state variable is introduced to capture reaction kinetics. Using mole fractions as the primary composition variables, the model enables a unified description of multiphase, multi-reaction liquid–solid systems, establishing an intrinsic coupling mechanism between chemical reactions and phase transformations. Compared with conventional phase-field approaches, this framework overcomes the limitations of classical models in treating chemically reactive phase transformations and offers a generalized description for complex reactive systems. Nevertheless, their work mainly focused on theoretical model development and demonstrated its applicability through the concurrent precipitation of Al_2_O_3_ and SiO_2_ inclusions. However, systematic studies on the parametric effects governing the growth and morphological evolution of individual inclusions, as well as the competitive growth of multiple oxide phases in molten steel, remain lacking. Furthermore, the role of interfacial anisotropy in the formation of dendritic inclusion morphologies has not yet been explored within this framework.

In this work, a numerical simulation framework is developed based on the reactive liquid–solid phase transformation theory proposed by Cao et al. [37], but reformulated in terms of molar concentrations. On this basis, the growth behavior of single Al_2_O_3_ inclusions is first investigated under isothermal and non-isothermal conditions, and the effects of key model parameters on inclusion growth are systematically analyzed by incorporating thermodynamic data for the formation of non-metallic oxide inclusions during molten steel refining. Furthermore, the simultaneous precipitation and competitive growth of Al_2_O_3_ and SiO_2_ composite inclusions are studied, with emphasis on how solute supply, diffusion, and phase-transformation kinetics regulate the resulting duplex morphology. Finally, interfacial energy anisotropy is incorporated into the phase-field evolution equation to examine the formation mechanism of dendritic inclusions. The remainder of this paper is organized as follows. Section 2 presents the governing equations and numerical implementation. Section 3 presents the simulation results and discussion, including the growth behavior of spherical inclusions, the formation and morphological evolution of dendritic inclusions, and the co-evolution of composite inclusions. Finally, Section 4 summarizes the main conclusions.

## 2. Methodology

### 2.1. Phase-Field Model

The evolution equations originally formulated in terms of mole fractions by Cao et al. [37] are reformulated in terms of concentrations in the present study. This modification facilitates the evaluation and verification of mass conservation on a volumetric basis, while providing a more direct and concise description of the reactive liquid–solid phase transformation process. The present model incorporates three coupled field variables: (i) the conserved *concentration* field c(x,t), a vector field with its components representing the molar concentrations of individual species; (ii) the non-conserved *order-parameter* field φ(x,t), representing the volume fractions of multiple solid phases that satisfy the local constraint $\sum \varphi_{i}<1$, where ∑ denotes the summation over all solid phases; (iii) the *temperature* field T(x,t). Based on the aforementioned framework and variable definitions, the dynamic behavior of the model is governed by the following coupled partial differential equations.

#### 2.1.1. Evolution of the Concentration Field

The system involves n chemical reactions associated with liquid–solid phase transformations and m species in total, among which $m^{\prime }$ correspond to solid phases. To describe the reaction pathways, each reaction is decomposed into two elementary steps via a *transition state*. For example, a reaction of the form A + B → C + D is represented as A + B → M and M → C + D, where M denotes the transition state. Consequently, n reactions correspond to 2n elementary reactions. The concentration $c=\left(c_{1},c_{2},\ldots ,c_{m+n}\right)$ is a column vector that contains the concentrations of m *dissolved* species in the liquid phase together with n transition states (one for each chemical reaction). It should be noted that the m dissolved species contained in c consist of ($m-m^{\prime }$) species in the liquid phase and $m^{\prime }$ species corresponding to molten counterparts of solid phases to be precipitated. The evolution of c(x,t) is governed by the following conservative equation that accounts for solute diffusion, chemical reaction, and mass consumption due to phase transformation, which is given by Ref. [37]:

(1) $$\frac{\partial c}{\partial t}=\lambda \cdot \xi +\nabla \cdot \left((1-\sum_{i=1}^{m^{\prime }}\varphi_{i})D\cdot \nabla c^{l}\right)+\rho^{M}\circ \frac{\partial \varphi }{\partial t},$$

where “·” denotes the dot product, “∇” is the gradient operator, and “∘” denotes the Hadamard (element-wise) product, meaning that the multiplication is performed component-wise between the corresponding entries of the vectors. The meaning of each term in Equation (1) is as follows.

The first term on the right-hand side of Equation (1) describes the chemical reaction, where λ is the second-order stoichiometric tensor where its first index corresponds to the m+n species in the concentration vector c, whereas the second index corresponds to the 2n elementary reactions. The term ξ is the reaction rate, a vector expressed as

(2) $$\begin{array}{c} \xi =-L_{c}\lambda^{T}\cdot \frac{\partial f\left(c\right)}{\partial c} \end{array}.$$

Here $L_{c}$ is the kinetic parameter for chemical reactions, the superscript “T” denotes the transpose of the tensor λ, and $f\left(c\right)$ is the chemical free energy density function given by

(3) $$\begin{array}{c} f\left(c\right)=c\cdot \mu^{\circ }+C_{R}T\sum_{j=1}^{m+n}\left[\left(\ln\left(\gamma_{j}\frac{c_{j}}{c^{\circ }}\right)-1\right)c_{j}\right]+\sum_{i=1}^{n}f_{i}^{a}e^{\kappa_{i}\frac{c_{m+i}}{c^{\circ }}} \end{array},$$

where $\mu^{\circ }$ is the standard chemical potential of the species in its reference state, $c^{\circ }$ is the standard concentration used to normalize the concentration $c_{j}$, $C_{R}$ is a constant related to the universal gas constant R, and $\gamma_{j}$ denotes the activity coefficient accounting for the deviation of the real solution from ideal behavior. The third term of Equation (3) describes the contribution of energetic barriers to the chemical free energy, where $f_{i}^{a}$ and $\kappa_{i}$ are constants associated with the activation energy barrier of the i-th reaction, and $c_{m+i}$ is the concentration of i-th transition state.

The second term in Equation (1) represents solute diffusion, with $D=diag\left(D_{11},D_{22},\ldots ,D_{\left(m+n)(m+n\right)}\right)$ as the material-dependent second-order diagonal diffusion tensor, where $D_{jj}$ denotes the diffusion coefficient of the j-th species and the off-diagonal components are zero, and $c^{l}$ as the molar concentration of species in the liquid phase. Diffusion is assumed to occur only in the liquid phase, while diffusion in solid phases is neglected. Note that $c^{l}$ is defined using the volume of liquid rather than the total volume as in c, and is thus related to the latter via $c^{l}=c/(1-\sum_{i=1}^{m^{\prime }}\varphi_{i})$.

The third term in Equation (1) represents the solute concentration consumed or released during the phase transformation. This term pertains solely to dissolved substances that are able to transform into the solid phase and is consequently set to zero for the rest of the calculation. The components of $\rho^{M}$ are defined as $\rho_{i}^{M}=\rho_{i}/M_{i}$, representing the molar density of the i-th species. In the numerical implementation, these component values are assembled into a vector $\rho^{M}$ for the purpose of component-wise array operations.

#### 2.1.2. Evolution of the Order-Parameter Field

The evolution of the order-parameter field φ(x,t) characterizes the kinetics of phase transformation, which is given by Ref. [37] as follows:

(4) $$\begin{array}{c} \frac{\partial \varphi }{\partial t}=L_{\varphi }\left(\nabla \cdot \left(\kappa^{\varphi }\cdot \nabla \varphi \right)-\frac{\partial f^{P}}{\partial \varphi }\right) \end{array}.$$

In Equation (4), the interface energy gradient term tends to smooth spatial variations in φ in order to minimize the interfacial energy. $L_{\varphi }$ is the phase-field kinetic coefficient governing the rate of interface migration. Notably, to account for the anisotropic characteristic of interfacial energy that was neglected in the original isotropic framework, we newly introduce an anisotropic second-order diagonal interfacial energy tensor $\kappa^{\varphi }$ in this work, expressed as

(5) $$\kappa^{\varphi }=diag\left(\kappa_{11}^{\varphi },\kappa_{22}^{\varphi },\ldots ,\kappa_{m^{\prime }m^{\prime }}^{\varphi }\right),\;\;\;\;\kappa_{ii}^{\varphi }={\left[\kappa_{\circ \left(i\right)}^{\varphi }\;\left(1+\epsilon_{i}cosm_{i}\theta_{i}\right)\right]}^{2},$$

where $\kappa_{\circ (i)}^{\varphi }$ is the gradient energy coefficient, $\epsilon_{i}$ denotes the anisotropy strength, $m_{i}$ represents the crystallographic symmetry, and $\theta_{i}$ is defined as the counterclockwise angle between the interface normal vector and the positive reference coordinate axis in the Cartesian coordinate system. $f^{P}$ in Equation (4) is the bulk free energy density, which, for each component $\varphi_{i}$, exhibits a double-well form when the other components of φ are fixed, given by Ref. [37], as follows:

(6) $$f^{p}=a_{0}+a_{1\left(i\right)}\varphi_{i}+a_{2\left(i\right)}{\varphi_{i}}^{2}+a_{3\left(i\right)}{\varphi_{i}}^{3}+a_{4\left(i\right)}e^{\zeta_{\left(i\right)}\left(\varphi_{l}-\varphi_{i}\right)}+a_{5\left(i\right)}\left[\frac{m^{\prime }-1}{m^{\prime }}e^{\zeta_{\left(i\right)}\left(\varphi_{i}-\varphi_{s}\right)}+\frac{1}{m^{\prime }}e^{\zeta_{\left(i\right)}\left(\hat{\varphi }-\varphi_{s}\right)}\right]$$

where $a_{1\left(i\right)}$, $a_{2\left(i\right)}$, …, $a_{5\left(i\right)}$, and $\zeta_{\left(i\right)}$ are coefficients associated with the i-th solid phase. Here, $\varphi_{l}=0$ and $\varphi_{s}=1$ represent the values of φ in the liquid and solid phases, respectively, and $\hat{\varphi }=\sum_{i=1}^{m^{\prime }}\varphi_{i}$ denotes the total solid fraction.

#### 2.1.3. Evolution of the Temperature Field

The evolution of the temperature field T(x,t) accounts for energy transport and conversion within the system as follows:

(7) $$\begin{array}{c} \rho c_{T}\frac{\partial T}{\partial t}=\nabla \cdot \left(\kappa^{T}\nabla T\right)-∆H_{r}\cdot \xi +∆H_{sol}\cdot (\rho^{M}\circ \frac{\partial \varphi }{\partial t}) \end{array}.$$

The volumetric heat capacity $\rho c_{T}(\varphi )$ is interpolated using $\left(1-\sum_{i=1}^{m^{\prime }}\varphi_{i}\right)*\rho_{l}*{c_{T}}_{l}+\sum_{i=1}^{m^{\prime }}\left(\varphi_{i}*\rho_{i}*{c_{T}}_{i}\right)$, where $\rho_{l}$ and ${c_{T}}_{l}$ are the mass density and specific heat capacity of the liquid solution, respectively, and $\rho_{i}$ and ${c_{T}}_{i}$ are those of the i-th solid species. The first term describes heat conduction within the system, where the effective thermal conductivity $\kappa^{T}$ is given by $\left(1-\sum_{i=1}^{m^{\prime }}\varphi_{i}\right)*\kappa_{l}^{T}+\sum_{i=1}^{m^{\prime }}\left(\varphi_{i}*\kappa_{i}^{T}\right)$. The second term represents the heat release/absorption from chemical reactions, with $∆H_{r}$ as the reaction enthalpy vector. The third term describes the heat effect associated with phase dissolution/precipitation, where $∆H_{sol}$ is the dissolution enthalpy vector.

### 2.2. Dimensionless Forms of Governing Equations

To facilitate the numerical implementation as well as the subsequent parametric and sensitivity analyses, the governing equations presented in Section 2.1 are rewritten in a fully dimensionless form using the following normalization parameters:

(8) $$\bar{l}=\frac{l}{l_{0}},\;\;\;\;\bar{c}=\frac{c}{c_{0}},\;\;\;\;\bar{t}=\frac{t}{t_{0}},\;\;\;\;\bar{T}=\frac{T}{T_{0}},$$

where $l_{0}$, $c_{0}$, $t_{0}$, and $T_{0}$ denote the characteristic length, concentration, time and temperature scales, respectively. Substituting Equation (8) into Equations (1), (4) and (7) and rearranging the terms, the following dimensionless governing equations are obtained:

(9) $$\frac{\partial \bar{c}}{\partial \bar{t}}=\lambda \cdot \left(-{\bar{L}}_{c}\lambda^{T}\right)\cdot \left[{\bar{\mu }}^{\circ }+{\bar{C}}_{R}\bar{T}\ln\left(\gamma \circ \frac{\bar{c}}{{\bar{c}}^{\circ }}\right)+\sum_{i=1}^{n}\frac{\kappa_{i}{\bar{f}}_{i}^{a}\delta_{m+i,j}}{{\bar{c}}^{\circ }}e^{\kappa_{i}\frac{{\bar{c}}_{m+i}}{{\bar{c}}^{\circ }}}\right]+\bar{\nabla }\cdot \left(\left(1-\sum_{i=1}^{m^{\prime }}\varphi_{i}\right)\bar{D}\cdot \bar{\nabla }{\bar{c}}^{l}\right)+{\bar{\rho }}^{M}\circ \frac{\partial \varphi }{\partial \bar{t}},$$

(10) $$\frac{\partial \varphi }{\partial \bar{t}}={\bar{L}}_{\varphi }\left(\bar{\nabla }\cdot \left({\bar{\kappa }}^{\varphi }\cdot \bar{\nabla }\varphi \right)-\frac{\partial {\bar{f}}^{P}}{\partial \varphi }\right),$$

(11) $$\frac{\partial \bar{T}}{\partial \bar{t}}=\frac{Rc_{0}}{\rho c_{T}}\left[\bar{\nabla }\cdot \left({\bar{\kappa }}^{T}\bar{\nabla }\bar{T}\right)-∆{\bar{H}}_{r}\cdot \bar{\xi }+∆{\bar{H}}_{sol}\cdot ({\bar{\rho }}^{M}\circ \frac{\partial \varphi }{\partial \bar{t}})\right],$$

where $\delta_{m+i,j}$ is the Kronecker delta and $\bar{\nabla }=l_{0}\nabla$. Table 1 summarizes the definitions of the main dimensionless parameters.

### 2.3. Numerical Implementation

The simulations are designed to investigate the local growth behavior of non-metallic inclusions after nucleation during molten steel refining, with particular emphasis on the coupled evolution of concentration, phase-field, and temperature fields in the vicinity of a single nucleation site. In the aluminum deoxidation system, alumina precipitation is governed by the reaction

(12) $$[\mathrm{Al}]+1.5[O]\;\to \;0.5{\mathrm{Al}}_{2}\;O_{3}(s).$$

The overall reaction is represented by two sequential elementary steps: [Al] + 1.5[O]$\;\to \;M^{A}$ and $M^{A}\to \;$0.5Al_2_O_3_(s). For this reaction system, λ is taken as

$$\left[\begin{array}{cc} \begin{array}{c} -1\\ -1.5\\ 1 \end{array} & \begin{array}{c} 0\\ 0\\ -1 \end{array}\\ 0 & 0.5 \end{array}\right].$$

To capture the precipitation process more explicitly, the second step is further interpreted as a two-stage process: the dissolved alumina is formed first, followed by their precipitation as solid Al_2_O_3_, expressed as $M^{A}\to \;$0.5Al_2_O_3_(L) and 0.5Al_2_O_3_(L) → 0.5Al_2_O_3_(s), respectively.

Simulations are performed in a two-dimensional square domain with a dimensionless side length of 1, discretized by a 1024 × 1024 uniform grid. This domain corresponds to a horizontal cross-section within the liquid steel bulk, away from the slag–steel interface and bottom stirring region. For this cross-section, radiative heat transfer is not considered, as the model focuses on the local precipitation behavior within the molten steel interior. Initially, aluminum and oxygen are uniformly dissolved in the liquid steel. The order-parameter field φ is initialized to 0.99 within a circular region centered at the domain center with a radius of 8 grid cells, representing a pre-existing oxide nucleus, and is initialized to zero elsewhere. The coupled evolution equations—Equations (9)–(11)—are solved using a finite-difference scheme. For spherical inclusion, spatial derivatives of the order-parameter field are evaluated using a second-order rotational Laplacian operator [37]. Time integration uses an explicit scheme with a constant time step $∆\bar{t}=0.0001$ up to a final dimensionless time of 1 (corresponds to a physical time of approximately 360 s). Zero-flux Neumann boundary conditions are applied to all variables via mirror extension. The dimensionless parameters and corresponding reference values are listed in Table 2.

The standard Gibbs free energy change $∆G^{{}^{\circ}}$ is formally expressed as the product $\lambda^{T}\cdot \mu^{\circ }$. When the temperature field is not coupled to phase evolution, a constant uniform temperature is assumed over the entire domain. $\bar{D}$ and γ are treated as scalars and take the same value for all components. The double-well potential function ${\bar{f}}^{p}$ takes the following form, with coefficients given approximately as

(13) $$f^{p}=-0.0144+1.15\varphi -0.084\varphi^{2}-16.7\varphi^{3}+0.0144e^{-80\varphi }+0.613e^{80\left(\varphi -1\right)}.$$

It should be noted that except for the Gibbs free energy change and intrinsic material properties (e.g., density and specific heat capacity), all other parameters are not taken from actual industrial data, but are selected for numerical demonstration and mechanistic analysis. Unless otherwise specified, these parameter values (Table 2) are used throughout the study to ensure consistency when analyzing the effects of parameter variations, and thermal evolution in the subsequent sections.

To further ensure that the numerical results are not affected by the choice of discretization parameters, time-step sensitivity, and grid-independence, benchmark tests are performed before the subsequent parametric studies. In the time-step sensitivity test (Table 3), the relative differences remain below 0.22% so long as the time step is below 0.0002. Consequently, a constant time step of $∆\bar{t}=0.0001$ is used in the baseline simulations.

A grid-independence test is then performed using different grid resolutions. For this test, the initialization of the order-parameter field is slightly different from that used in the baseline case. Specifically, φ is initialized to 0.99 in a square region with a side length of 0.125 at the domain center and is initialized to zero elsewhere, so that the initial mean value of φ remains the same for all grid resolutions. As shown in Table 4, the relative differences between adjacent grid resolutions decrease with grid refinement. When the grid resolution is increased from 1024 × 1024 to 2048 × 2048, the relative changes in the monitored quantities are no larger than 1.77%, indicating that the numerical results are virtually grid-independent. Therefore, a 1024 × 1024 grid is adopted in the baseline simulations to balance numerical accuracy and computational cost.

**Figure 1:**
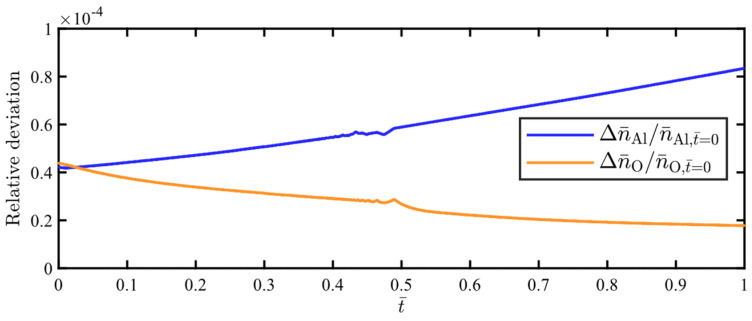
Relative deviations of total Al and O contents with dimensionless time for mass conservation verification.

Additionally, to verify the conservation property of the model during long-time simulations, the variations in the total amounts of Al and O in the system were evaluated. As shown in Figure 1, the relative deviations of the total Al and O contents remain within the order of 10^−4^ throughout the simulation, indicating that the established model exhibits good mass conservation during long-time integration.

## 3. Results and Discussion

### 3.1. Growth of a Single Spherical Inclusion

#### 3.1.1. Isothermal Precipitation

During the steel refining process, the temperature variation within the time scale of inclusion precipitation is usually negligible. Therefore, the baseline simulations in this section are conducted under isothermal conditions, focusing on the coupled evolution of the concentration and phase fields.

**Figure 2:**
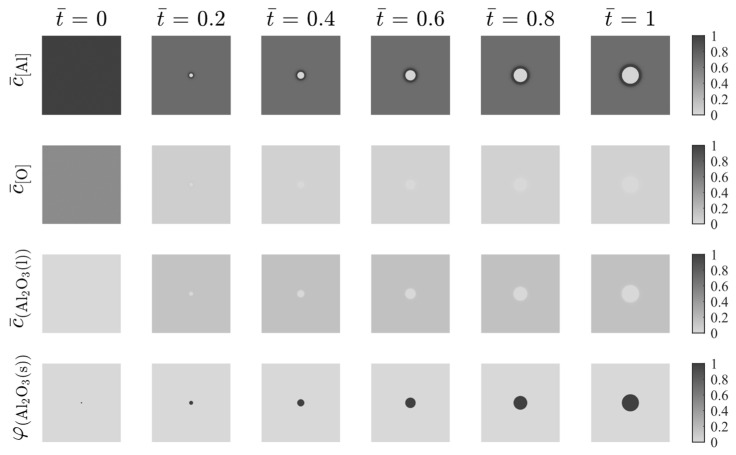
Snapshots of simulated precipitation process of a single Al_2_O_3_ spherical inclusion, showing the spatial distributions of the concentration fields and the order-parameter field.

**Figure 3:**
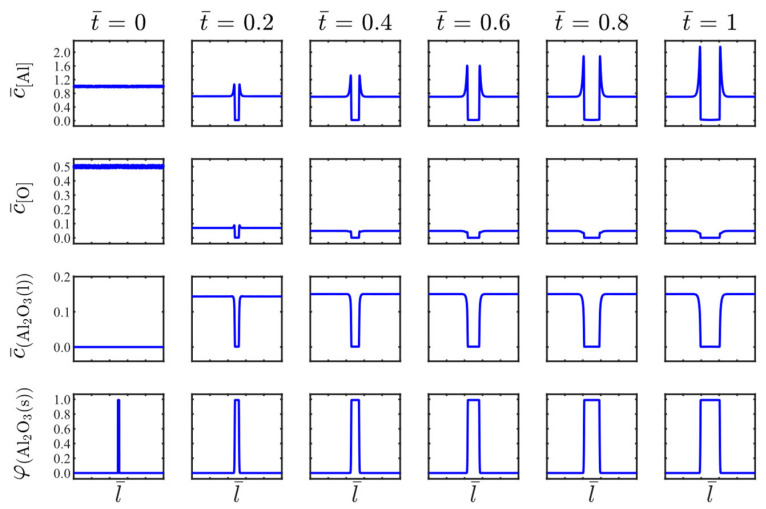
Profiles of the concentration fields and the order-parameter field along the horizontal centerline of the computational domain at various dimensionless times (corresponding to Figure 2).

The spatiotemporal evolution of the governing physical fields and their corresponding spatial profiles at selected dimensionless times ($\bar{t}$ = 0~1.0) are presented in Figure 2 and Figure 3. During the initial stage ($\bar{t}$ = 0~0.2), the concentrations of ${\bar{c}}_{\left[Al\right]}$ and ${\bar{c}}_{\left[O\right]}$ decrease significantly, while ${\bar{c}}_{\left(Al2O3\left(L\right)\right)}$ increases markedly, indicating that the system is in a highly reactive and nonequilibrium state during this period. Once Al_2_O_3_(L) is formed, the pre-existing oxide nucleus begins to grow through the liquid-to-solid phase transformation process. As time progresses, the alumina inclusion develops under the combined effects of interfacial energy and bulk free energy of the order parameter $\varphi_{\left(Al2O3\left(s\right)\right)}$. The liquid–solid interface propagates outward while $\varphi_{\left(Al2O3\left(s\right)\right)}$ at the interface increases continuously, eventually forming an approximately circular solid inclusion. With the continuous growth of the solid phase, the surrounding aluminum solute is progressively rejected into the liquid region, leading to solute enrichment near the interface and the formation of a characteristic *bimodal* distribution. Figure 2 clearly demonstrates the transport pathway of elements from the molten steel toward the inclusion. Meanwhile, the spatial profile of $\varphi_{\left(Al2O3\left(s\right)\right)}$ evolves from an initially sharp peak ($\bar{t}$ = 0~0.2), reflecting the localized initial nucleus, to a plateau-like distribution ($\bar{t}$ = 0.4~1.0), indicating the establishment of a stable growth regime.

From a kinetic perspective, the inclusion growth undergoes a clear transition from interface-reaction control to diffusion control. In the early stage ($\bar{t}$ = 0~0.2), the system is far from thermodynamic equilibrium with a high chemical driving force. The interfacial reaction rate is much faster than the solute diffusion rate, so the inclusion nucleates and grows rapidly. As solute near the interface is consumed and rejected solute accumulates to form a diffusion boundary layer, the mass transfer resistance in the liquid phase increases gradually. The rate-limiting step of growth shifts from interfacial reaction to bulk diffusion, and the growth rate slows down accordingly, finally entering a quasi-steady growth regime after $\bar{t}$ = 0.4.

The nearly circular morphology is a result of interfacial energy minimization. Under isothermal, quiescent and isotropic conditions, the interfacial energy is uniform in all directions. The system spontaneously evolves into a spherical shape (circular in 2D) to reduce the total interfacial free energy, which is the typical equilibrium morphology of oxide inclusions in static molten steel.

#### 3.1.2. Non-Isothermal Precipitation

In this section, the coupled evolution of the temperature field, concentration field, and order-parameter field is investigated in an isolated system without convection and radiation. The initial dimensionless temperature is set uniformly to 1 throughout the computational domain. The heat source in the system originates from the exothermic chemical reaction and the latent heat released during solute precipitation. Within this framework, the influence of temperature field evolution on the inclusion precipitation is examined. A smaller time step of 0.000025 is used here.

**Figure 4:**
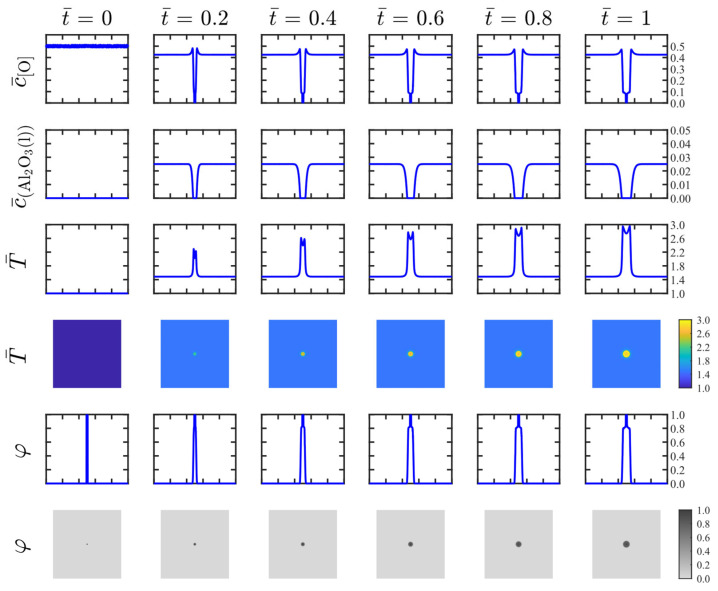
Spatiotemporal evolution of the concentration fields, the temperature field and the order-parameter field.

The results with the coupled evolution of the temperature field are presented in Figure 4. The chemical reaction mainly proceeds in the early stage ($\bar{t}$ = 0~0.2) and releases a large amount of heat, leading to a significant temperature rise in the entire domain. The elevated temperature consequently increases the Gibbs free energy change in the reaction, which considerably suppresses the forward reaction process. Compared with the results in Figure 3, the concentration of liquid alumina solute ${\bar{c}}_{\left(Al2O3\left(L\right)\right)}$ decreases to approximately 0.026, so that the alumina solute cannot be completely transformed into the solid phase during phase transformation. As a result, the inclusion growth rate is significantly reduced, and the final inclusion size is notably smaller than that obtained under the isothermal condition shown in Figure 2. The local peaks in the temperature field reflect the contribution of latent heat released from phase transformation to the temperature increase in the system.

To evaluate the influence of heat convection on inclusion precipitation, the temperature evolution equation is modified by introducing the convection term as follows:

(14) $$\begin{array}{c} \rho c_{T}(\frac{\partial T}{\partial t}+u\cdot \nabla T)=\nabla \cdot \left(\kappa^{T}\nabla T\right)-∆H_{r}\cdot \xi +∆H_{sol}\cdot (\rho^{M}\circ \frac{\partial \varphi }{\partial t}) \end{array}.$$

Equation (14) is subsequently nondimensionalized with characteristic reference quantities, and the dimensionless flow velocity is set to $\bar{u}$ = (0.2,0.2) in this case. To decouple thermal-transport and solute-transport effects, only heat convection is considered here, and solute advection will be investigated in our future work.

**Figure 5:**
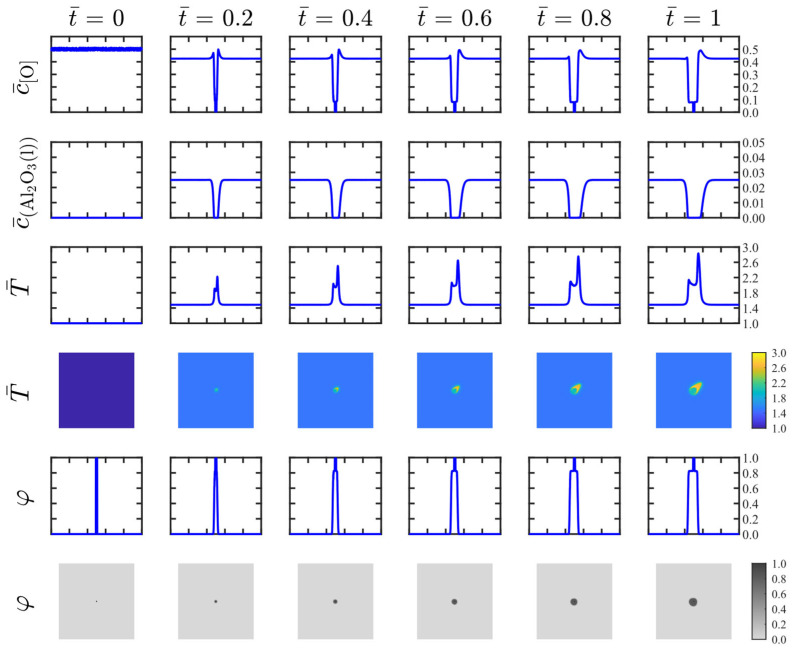
Spatiotemporal evolution of the concentration fields, the temperature field and the order-parameter field with thermal convection.

The spatiotemporal evolution of each field under thermal convection is presented in Figure 5. Compared with the pure heat conduction case in Figure 4, the introduction of thermal convection induces notable changes in temperature distribution, and further affects solute evolution and inclusion growth. With the advection effect of fluid flow, heat accumulated in the inclusion region is continuously transported outward. As a result, the overall temperature at the inclusion is lower than that in the pure conduction case, and the originally symmetric high-temperature region is stretched along the flow direction (diagonal direction of the x and y axes), forming an inclined elliptical high-temperature zone with expanded coverage. The local temperature peaks still correspond to the latent heat release from phase transformation, which superimposes with chemical reaction heat to raise the local temperature, while convection weakens the degree of heat concentration and flattens the temperature distribution. The oxygen consumption in the central region is slightly intensified, with a reduced concentration peak at the left interface and a slightly elevated peak at the right interface. For the liquid alumina concentration, the gradient becomes steeper on the left side of the central region and gentler on the right side. This distribution feature directly corresponds to the shift in the temperature field: the overall temperature in the central region decreases, the temperature drops at the left interface, and the high-temperature zone expands on the right side. Compared with the centrally symmetric morphology under pure heat conduction, both the left and right interfaces of the solid inclusion shift toward the left (upstream side) under thermal convection, breaking the original central symmetry.

This indicates that during inclusion growth, inconsistent temperature variations and differentiated solute supply across the interface lead to uneven interface advancement velocities. As a result, the center of the inclusion deviates from the original nucleation site and shifts accordingly. In addition, the temperature rise induced by the exothermic reaction essentially forms a self-inhibiting thermal feedback mechanism. The released heat reduces the local thermodynamic driving force in real time, which naturally restricts the rapid growth of inclusions in the early precipitation stage. This inherent thermal self-regulation effect is a typical coupling feature of the deoxidation–precipitation process, which cannot be captured in an isothermal simulation framework.

The temperature rise exhibited in both the pure conduction and thermal convection cases in this section does not represent the actual temperature variation in industrial steel refining processes, and the magnitude of temperature change under real operating conditions is considerably smaller. In this study, a relatively high characteristic concentration is adopted to amplify the thermal feedback induced by the exothermic deoxidation reaction, which facilitates the investigation of the intrinsic coupling mechanism between temperature evolution and inclusion growth, as well as the modulation effect of thermal convection on such coupling.

#### 3.1.3. Parametric Analysis

**Figure 6:**
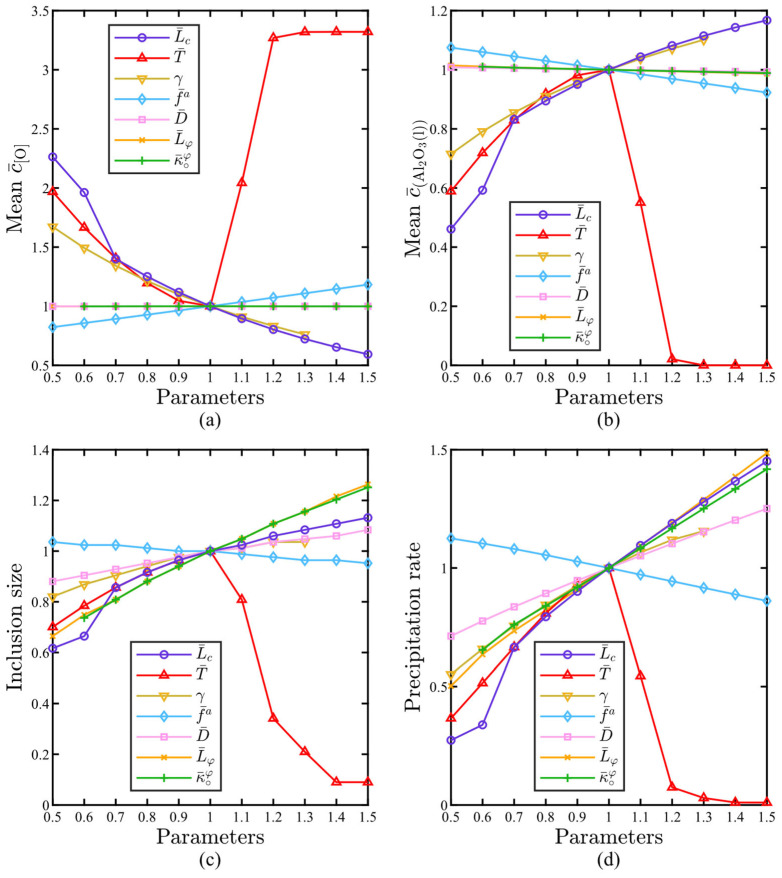
Response relationships between normalized parameters and normalized key indicators of melt composition and inclusion precipitation at ${\bar{L}}_{c}$ = 0.2: (**a**) oxygen concentration ${\bar{c}}_{\left[O\right]}$; (**b**) dissolved alumina concentration ${\bar{c}}_{\left(Al2O3\left(L\right)\right)}$; (**c**) inclusion size; (**d**) precipitation rate of inclusion.

Based on the case of spherical inclusion growth under constant temperature presented in Section 3.1.1, the influences of different parameters on the simulation results are further investigated. Accordingly, a parametric analysis is conducted to investigate the influences of seven representative dimensionless parameters on the reactive precipitation behavior of inclusions. The corresponding response relationships are shown in Figure 6.

Increasing the chemical reaction kinetic parameter ${\bar{L}}_{c}$ and activity coefficient γ promotes the forward reaction and inclusion growth, whereas a larger activation energy barrier constant ${\bar{f}}^{a}$ inhibits both processes. The temperature effect is distinctly non-monotonic, arising from the competition between the temperature dependence of the standard Gibbs free-energy change and the entropy-related contribution embedded in the thermodynamic description. When $\bar{T}<1$, the gradual decrease in the average oxygen concentration with increasing temperature indicates that the mixing-entropy contribution dominates over the temperature dependence of the reaction free energy. As $\bar{T}$ increases further beyond 1.0, the average oxygen concentration rises rapidly, while the alumina concentration drops sharply and approaches zero, indicating that the temperature dependence of the free-energy change becomes dominant. Therefore, $\bar{T}=1$ corresponds to the temperature condition that maximizes the precipitation tendency within the present simulations.

In addition, increasing the diffusion coefficient $\bar{D}$, phase-field kinetic coefficient ${\bar{L}}_{\varphi }$, and gradient energy coefficient ${\bar{\kappa }}_{\circ }^{\varphi }$ generally facilitates inclusion growth within the parameter range examined in Figure 5. It should be noted, however, that ${\bar{\kappa }}_{\circ }^{\varphi }$ is expected to have an optimal range rather than increasing the growth rate indefinitely. When ${\bar{\kappa }}_{\circ }^{\varphi }$ is relatively small, the interfacial energy is insufficient to sustain growth, leading to stagnation. Conversely, at relatively large ${\bar{\kappa }}_{\circ }^{\varphi }$, the interface thickness increases significantly, resulting in enhanced diffusive smearing. As a consequence, the initially prescribed solid region is rapidly dispersed before it can develop into a stable growing inclusion, thereby inhibiting subsequent inclusion growth.

Unless otherwise specified, all parameters are varied within 0.5~1.5 times their respective reference values. The reference temperature of 1873 K corresponds to the typical operating temperature during ladle refining. The temperature range considered in this study extends beyond the fluctuations encountered in practical steelmaking in order to fully capture the non-monotonic precipitation behavior of oxide inclusions with temperature, rather than to quantitatively reproduce industrial processing conditions. The phase-field mobility and gradient energy coefficient are phenomenological parameters that cannot be directly determined experimentally. Their reference values are selected based on previous phase-field studies and calibrated to produce physically reasonable interfacial evolution. In addition, identical activity coefficients and diffusion coefficients are assigned to all species to minimize the influence of differences in material properties, thereby allowing the effects of individual controlling parameters on inclusion precipitation and growth to be examined more clearly.

### 3.2. Formation and Co-Evolution of Composite Inclusions

Experimental studies have shown that non-metallic inclusions in steel often exhibit complex composite structures rather than simple single-phase morphologies. For instance, Wu et al. [39] reported that Al_2_O_3_-TiO_x_-MgO composite inclusions with multi-layered and mosaic structures can form in Al-Ti deoxidized and Mg-treated steels, while Liu et al. [40] revealed that Al_2_O_3_-MnS composite inclusions play a critical role in localized corrosion initiation. These studies, based on combined SEM and EDS characterization, highlight the coexistence and interaction of multiple phases within individual inclusions, as well as their significant influence on microstructure evolution and material performance.

Consequently, the present study considers the simultaneous formation of different oxide phases within a unified numerical framework under isothermal conditions. In the following, alumina (Al_2_O_3_) and silica (SiO_2_) are taken as representative components to investigate the formation and co-evolution of composite inclusions. For the reaction [Si] + 2[O] → SiO_2_ (s), the standard Gibbs free energy change $∆G^{{}^{\circ}}$ is given by (−576,440 + 218.2T) J/mol [38]. Here, ${\bar{c}}_{\left[Al\right]}$, ${\bar{c}}_{\left[Si\right]}$, and ${\bar{c}}_{\left[O\right]}$ are all set to 1. The time step is set to 0.00002.

**Figure 7:**
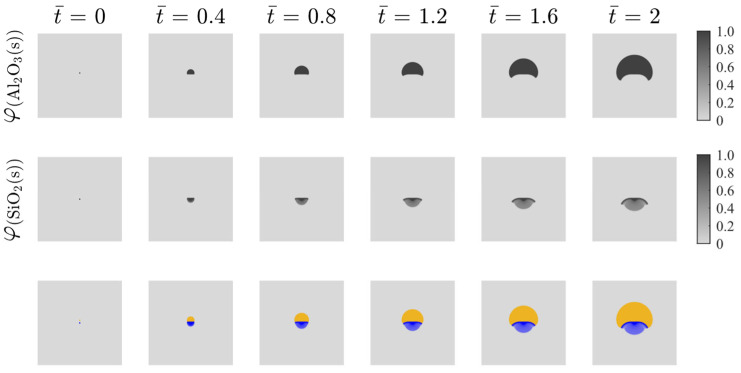
Snapshots of simulated concurrent precipitation of Al_2_O_3_ and SiO_2_ inclusions, showing the evolution of order parameters $\varphi_{\left(Al2O3\left(s\right)\right)}$ (**top row**), $\varphi_{\left(SiO2\left(s\right)\right)}$ (**middle row**), and overall morphology (**bottom row**).

The spatial evolution of the order parameters for Al_2_O_3_(s) and SiO_2_(s) is shown in Figure 7. As time progresses, both inclusions grow within the computational domain. However, due to differences in the Gibbs free energy changes in the corresponding reactions, the resulting solute concentrations differ. Consequently, the growth rate of the Al_2_O_3_ phase is significantly higher than that of SiO_2_, leading to a larger occupied region at later stages. At $\bar{t}=1.0$, Al_2_O_3_ forms a relatively large inclusion, whereas SiO_2_ remains confined to a smaller region in its vicinity. Because the solute concentration of SiO_2_ is insufficient to fully transform into the solid phase, lower order parameter values are observed in the surrounding region (light gray areas in Figure 7). These results demonstrate that the present modeling framework is capable of capturing the simultaneous precipitation and morphological evolution of multiple oxide phases. The distinct growth behaviors of Al_2_O_3_ and SiO_2_ are primarily attributed to differences in their thermodynamic driving forces and associated kinetic parameters.

**Figure 8:**
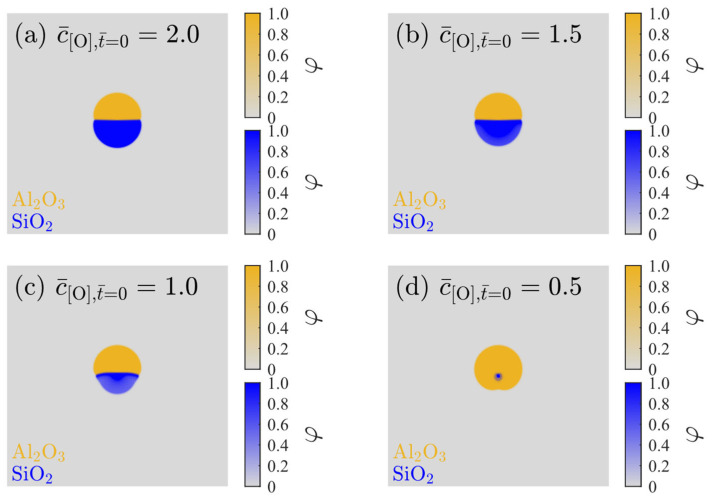
Morphological evolution of Al_2_O_3_ (yellow) and SiO_2_ (blue) inclusions under different initial oxygen concentrations ${\bar{c}}_{\left[O\right]}$: (**a**) 2.0, (**b**) 1.5, (**c**) 1.0, and (**d**) 0.5.

The effect of initial oxygen concentration on the co-evolution of Al_2_O_3_ and SiO_2_ inclusions is presented in Figure 8. At a relatively high oxygen concentration (${\bar{c}}_{\left[O\right],\bar{t}=0}=2.0$), both Al_2_O_3_ and SiO_2_ phases can fully develop, forming a composite inclusion with comparable spatial extents. As the oxygen concentration decreases to 1.5 and 1.0, the growth of the SiO_2_ phase is progressively suppressed, leading to a reduced volume fraction and a more localized distribution adjacent to the Al_2_O_3_ inclusion. When the oxygen concentration is further reduced to 0.5, the SiO_2_ phase can no longer sustain continuous growth and only a small remnant region is observed, indicating that the thermodynamic driving force is insufficient for its complete phase transformation. In contrast, the Al_2_O_3_ phase remains dominant across all cases due to its stronger thermodynamic driving force. These results demonstrate that the initial oxygen concentration plays a critical role in regulating the competitive growth of duplex oxide inclusions. A sufficiently high oxygen level favors the formation of composite inclusions, whereas lower oxygen concentrations shift the system toward Al_2_O_3_-dominated morphologies by suppressing the precipitation of SiO_2_.

**Figure 9:**
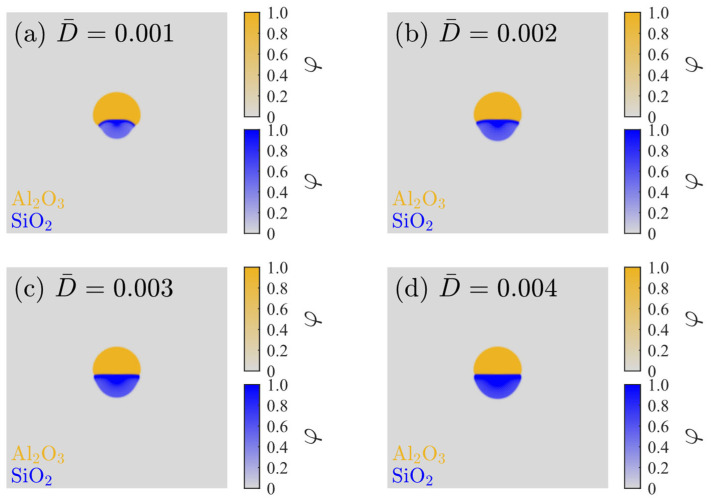
Morphological evolution of Al_2_O_3_ (yellow) and SiO_2_ (blue) inclusions under different diffusion coefficient $\bar{D}$ at ${\bar{c}}_{\left[O\right]}=1.0$: (**a**) 0.001, (**b**) 0.002, (**c**) 0.003, and (**d**) 0.004.

Figure 9 illustrates the effect of diffusion on the morphology of Al_2_O_3_-SiO_2_ duplex inclusions. In the present model, the diffusion coefficient mainly controls the transport of dissolved Al_2_O_3_ and SiO_2_ species after their formation by chemical reactions, rather than directly altering the reaction process. With increasing $\bar{D}$, these dissolved oxide species can be transported more efficiently toward the growing solid–liquid interface and subsequently transformed into the corresponding solid phases. As a result, both oxide phases grow more sufficiently, leading to a clearer and more stable duplex inclusion morphology.

**Figure 10:**
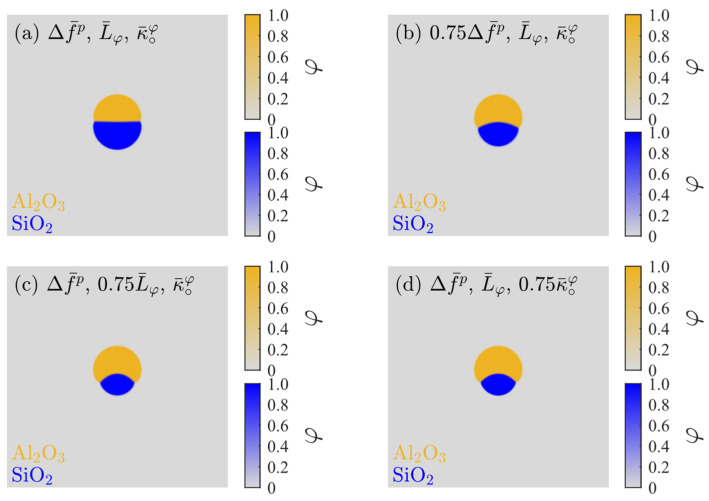
Effect of thermodynamic and kinetic parameters on the competitive growth between Al_2_O_3_ (yellow) and SiO_2_ (blue) inclusions at ${\bar{c}}_{\left[O\right]}=2.0$. (**a**) Reference case with baseline parameters; (**b**) reduced thermodynamic driving force of SiO_2_ (0.75$∆{\bar{f}}^{p}$); (**c**) reduced phase-field kinetic coefficient of SiO_2_ (0.75${\bar{L}}_{\varphi }$); and (**d**) reduced gradient energy coefficient of SiO_2_ (0.75${\bar{\kappa }}_{\circ }^{\varphi }$).

To further elucidate the origin of the competitive growth behavior between different oxide phases, a set of simulations was performed by selectively modifying the thermodynamic driving force, interface kinetics, and interfacial energy of the SiO_2_ phase (Figure 10). Here, $∆{\bar{f}}^{p}$ denotes the free energy density difference between the two stable states of the double-well potential, representing the thermodynamic driving force for phase transformation. As shown in Figure 10, reducing these parameters by the same proportion (0.75) leads to a pronounced suppression of SiO_2_ growth in all cases, indicating that thermodynamic driving force, interface kinetics, and interfacial energy all play essential roles in the formation kinetics of duplex inclusions. The reduction in $∆{\bar{f}}^{p}$ weakens the thermodynamic tendency for phase transformation, while decreasing ${\bar{L}}_{\varphi }$ directly slows the interface migration rate. In contrast, reducing ${\bar{\kappa }}_{\circ }^{\varphi }$ modifies the interfacial energy contribution and curvature-related stabilization, which affects the morphology and persistence of the SiO_2_ phase during competitive growth.

The final morphology of Al_2_O_3_-SiO_2_ duplex inclusions is governed by two coupled factors: the supply of transformable oxide solutes and the kinetics of liquid–solid transformation. The initial oxygen concentration, diffusion coefficient, and thermodynamic driving force mainly determine the amount and spatial availability of dissolved Al_2_O_3_ and SiO_2_ species, either by promoting their formation or by enhancing their transport to the growing interface. By contrast, the phase-field kinetic coefficient controls how efficiently these dissolved oxide species are converted into solid phases through interface propagation. Thus, the competitive morphology results from the balance between oxide-solute supply and transformation kinetics.

In practical steel refining processes, multiphase composite inclusions are a more prevalent form. Their phase composition, size and spatial structure not only affect the control of melt cleanliness, but also further influence the subsequent processing properties and service performance of steel products. Directionally regulating the competitive relationship between different oxides by adjusting process parameters, such as initial solute concentration and refining temperature, to achieve precise control over the morphology and phase fraction of composite inclusions has clear engineering guiding value for optimizing inclusion characteristics and improving the comprehensive properties of steel.

### 3.3. Formation of Dendritic Inclusion

In previous numerical simulations, an isotropic interfacial energy was assumed, leading to inclusions with ideal spherical morphologies. However, in practical steelmaking systems, alumina inclusions exhibit a variety of complex non-spherical morphologies. Ende et al. [41] experimentally observed polyhedral Al_2_O_3_ inclusions, while Fuseini et al. [42] further reported coral-like dendrites, plate-like dendrites, and angular clusters. Fundamentally, due to crystallographic symmetry, the interfacial energy is orientation-dependent, which constitutes the primary physical mechanism governing the formation of non-spherical and dendritic inclusion morphologies. Accordingly, the interfacial anisotropy model described by Equation (5) is introduced in this section to systematically investigate the formation mechanisms and morphological evolution of dendritic structures within the present numerical framework. Since this section focuses on the qualitative morphological characteristics of dendritic inclusions, the simulations are performed on a 128 × 128 grid with $∆\bar{t}=0.0002$. These simulations are intended to reveal the characteristic dendritic growth mode and morphological evolution.

**Figure 11:**
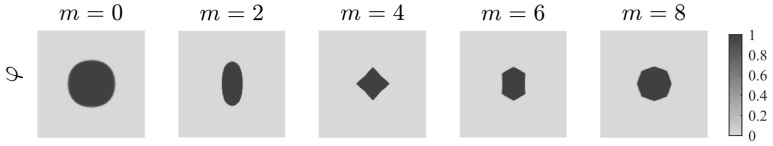
Final spatial morphologies of simulated Al_2_O_3_ inclusion at different anisotropy orders m, represented using the order parameters (ϵ=0 for m=0; ϵ=0.3 for all m>0).

**Figure 12:**
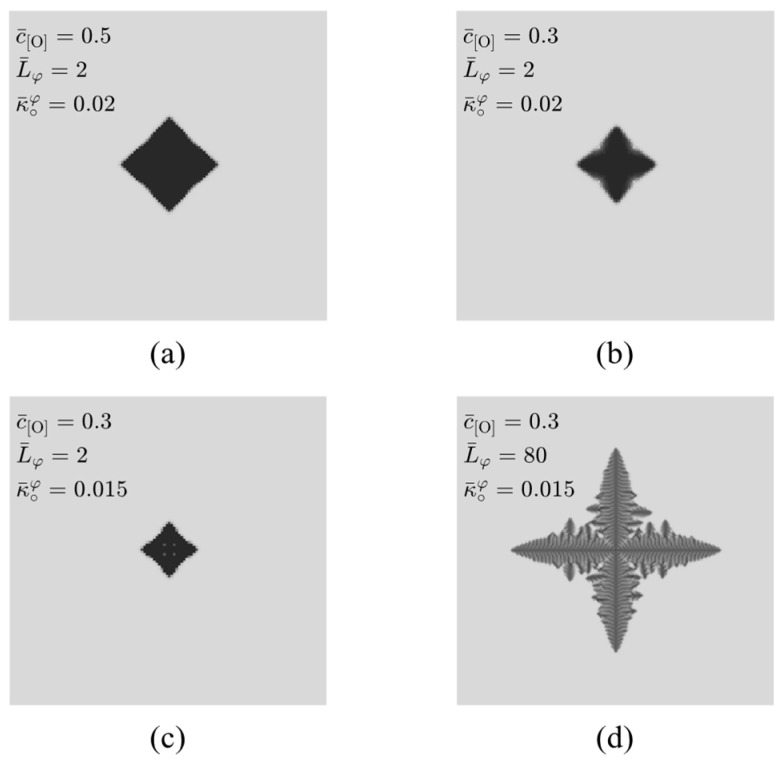
Morphological evolution of Al_2_O_3_ inclusion under different initial oxygen concentration ${\bar{c}}_{\left[O\right]}$, phase-field kinetic coefficient ${\bar{L}}_{\varphi }$, and gradient energy coefficient ${\bar{\kappa }}_{\circ }^{\varphi }$ at anisotropy strength ϵ=0.3, represented using the order parameters: (**a**) ${\bar{c}}_{\left[O\right]}=0.5$, ${\bar{L}}_{\varphi }=2$, ${\bar{\kappa }}_{\circ }^{\varphi }=0.02$; (**b**) ${\bar{c}}_{\left[O\right]}=0.3$, ${\bar{L}}_{\varphi }=2$, ${\bar{\kappa }}_{\circ }^{\varphi }=0.02$; (**c**) ${\bar{c}}_{\left[O\right]}=0.3$, ${\bar{L}}_{\varphi }=2$, ${\bar{\kappa }}_{\circ }^{\varphi }=0.015$; (**d**) ${\bar{c}}_{\left[O\right]}=0.3$, ${\bar{L}}_{\varphi }=80$, ${\bar{\kappa }}_{\circ }^{\varphi }=0.015$.

As depicted in Figure 11, under a fixed anisotropy strength ϵ=0.3, increasing the anisotropy order m significantly alters the final morphology of the inclusion. The perfectly spherical particle under isotropic conditions progressively develops obvious faceting and symmetrical dendritic edges, clearly demonstrating the essential role of orientation-dependent interfacial energy in producing realistic non-spherical alumina inclusion morphologies. As shown in Figure 12a–c, variations in the initial oxygen concentration significantly influence the growth behavior. A relatively high oxygen concentration results in compact, faceted morphologies, indicating that interfacial stabilization dominates under these conditions. In contrast, reducing the oxygen concentration promotes the development of dendritic features, as the system enters a regime where interfacial instability can be sustained. The effect of the phase-field kinetic coefficient is illustrated in Figure 12d, where a larger kinetic coefficient leads to pronounced dendritic branching with well-developed primary and secondary arms. This behavior suggests that increased interface mobility enhances the amplification of perturbations along the interface, thereby accelerating the transition from faceted growth to a dendritic morphology.

Figure 11 and Figure 12 indicate that the formation of dendritic alumina inclusions is governed by the combined effects of multiple parameters. The anisotropy strength and symmetry order together determine the preferred growth directions, while the gradient energy coefficient controls the overall interfacial stability and characteristic length scale. A lower initial oxygen concentration leads, through chemical reactions, to a reduced solute concentration of alumina and a growth driving force within a favorable range for dendritic growth. A larger phase-field kinetic coefficient promotes interfacial instability, thereby facilitating dendritic growth.

**Figure 13:**
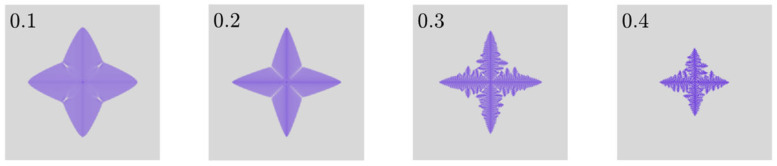
Morphological evolution of precipitated Al_2_O_3_ inclusion under different anisotropy strength ϵ at initial oxygen concentration ${\bar{c}}_{\left[O\right]}=0.3$, phase-field kinetic coefficient ${\bar{L}}_{\varphi }=80$, and gradient energy coefficient ${\bar{\kappa }}_{\circ }^{\varphi }=0.015$, represented using the order parameters.

Increasing the anisotropy strength ϵ (defined in Equation (5)) gradually transforms the alumina inclusion from a weakly faceted particle into a well-developed dendritic structure (Figure 13). At a relatively low anisotropy strength, the interface exhibits only a mild directional preference, resulting in rounded or slightly faceted shapes. As the anisotropy strength increases, pronounced growth along preferred crystallographic directions emerges, leading to distinct primary arms. A further increase in anisotropy sharpens the dendrite tips, indicating stronger orientation selection.

Mechanistically, increasing the anisotropy strength enhances the directional dependence of the interfacial energy, promoting the formation of protrusions at the interface. These protrusions extend into the supersaturated liquid and receive a higher local solute supply, leading to faster growth along those directions, namely, a manifestation of diffusion-limited instability. Thus, anisotropy determines the preferred growth directions, while the diffusion field amplifies the protrusions once they are formed.

**Figure 14:**
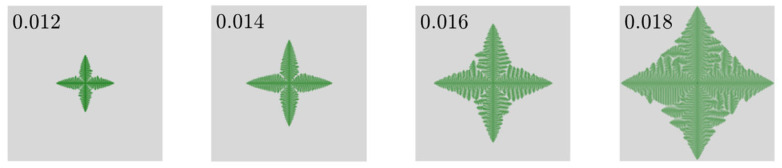
Morphological evolution of precipitated Al_2_O_3_ inclusion under different gradient energy coefficient ${\bar{\kappa }}_{\circ }^{\varphi }$ at initial oxygen concentration ${\bar{c}}_{\left[O\right]}=0.3$, phase-field kinetic coefficient ${\bar{L}}_{\varphi }=80$, and anisotropy strength ϵ=0.3, represented using the order parameters.

The influence of the gradient energy coefficient ${\bar{\kappa }}_{\circ }^{\varphi }$ (defined in Equation (5)) on dendritic morphology is illustrated in Figure 14. As the gradient energy coefficient increases, the overall size of the inclusion gradually increases, while the primary dendrite arms become more pronounced and secondary branching develops more extensively. Increasing the gradient energy coefficient enlarges the characteristic interfacial length scale and broadens the diffuse interface. As a result, the advancing dendrite tips can collect solute from a wider surrounding region, sustaining preferential growth at the tips. Meanwhile, the neighboring regions become relatively solute-depleted, which enhances the concentration gradient along the interface and facilitates the amplification of interfacial perturbations, eventually promoting the formation of secondary branches. Therefore, larger values of ${\bar{\kappa }}_{\circ }^{\varphi }$ promote the formation of larger and more complex dendritic structures with enhanced branching, highlighting the important role of the gradient energy coefficient in controlling the characteristic scale and morphological evolution of dendritic inclusions.

### 3.4. Model Assumptions and Applicability

To focus on the coupling between chemical reactions, solute diffusion, and phase transformation during oxide precipitation, several simplifying assumptions are adopted in the present model.

The first assumption is that solute diffusion is considered only in the liquid phase. This approximation is reasonable because oxide inclusions nucleate and grow in molten steel, while atomic diffusion in the solid oxide is several orders of magnitude lower than that in the liquid and therefore has a negligible influence on the overall precipitation kinetics. Consequently, the growth behavior is primarily controlled by mass transport in the surrounding liquid.

The second assumption is that fluid flow, natural convection, electromagnetic stirring, and inclusion movement are neglected. The objective of the present work is to investigate the intrinsic coupling between reaction kinetics, diffusion, interfacial evolution, and thermodynamic driving force. The introduction of fluid flow would mainly modify the external mass transport surrounding inclusions without changing the governing mechanisms incorporated in the present framework. Therefore, the current model is applicable to situations where diffusion dominates local transport or where the influence of convection can be treated separately.

In addition, the thermophysical properties used in the simulations are assumed to be constant within the investigated temperature range, and the computational domain is treated as an isolated representative volume without considering interactions among multiple moving inclusions. These assumptions improve model tractability while retaining the essential physics governing inclusion precipitation and growth, but also define the current scope of applicability of the present framework.

In addition to the physical assumptions discussed above, the predictive capability of the present model is subject to several limitations. Although the proposed framework successfully captures the coupled reaction–diffusion–phase transformation behavior of oxide inclusions and reproduces characteristic morphologies reported in experiments, the current validation remains primarily qualitative. The comparisons presented in this study focus on precipitation mechanisms, morphological evolution, and the effects of key controlling parameters. More comprehensive quantitative validation, including inclusion size, growth rate, phase fraction, and spatial distribution, through dedicated experiments or thermodynamic calculations, is still required to further assess the predictive capability of the model.

Furthermore, all simulations were performed in a two-dimensional computational domain. While this simplification substantially reduces computational cost and enables systematic parametric investigations, actual inclusion growth occurs in three-dimensional molten steel. Consequently, the present model cannot fully represent three-dimensional interfacial evolution, out-of-plane solute diffusion, dendritic branching, or competitive growth between Al_2_O_3_ and SiO_2_ inclusions. Extending the present framework to three-dimensional simulations will therefore be an important direction for future work.

## 4. Conclusions

In this study, the precipitation and growth behavior of alumina inclusions in molten steel are systematically investigated within a phase-field modeling framework. The governing equations largely follow the liquid–solid phase transformation model previously established by Cao et al. [37]. On this basis, the present work extends and improves the original model in the following three aspects: (1) the concentration evolution equation is reformulated using molar concentration instead of mole fraction; (2) interfacial energy anisotropy is incorporated into the order-parameter evolution equation; (3) thermal convection is additionally considered in Section 3.1.2 to examine its effect on inclusion growth. With these improvements, we establish a general numerical simulation framework for reactive liquid–solid phase transformations and apply it to the study of inclusion precipitation during the deoxidation stage in steel refining, extending the numerical investigations that were lacking in the previous work. The specific cases examined include the following: (1) the growth behavior of a single spherical inclusion under isothermal and non-isothermal conditions; (2) the morphological evolution of composite inclusions; and (3) the formation of dendritic inclusions. The main conclusions are as follows:

Inclusion growth is governed by the coupled effects of reaction kinetics, thermodynamic driving force, and interfacial dynamics. Temperature evolution introduces thermal feedback through reaction heat and latent heat, which modifies the thermodynamic driving force and suppresses inclusion growth. The temperature effect is found to be non-monotonic, with inclusion growth reaching a maximum within a specific temperature range in the present model. When thermal convection is present, the high-temperature zone is stretched along the flow direction into an inclined elliptical distribution, and the center of the solid inclusion shifts toward the upstream side, breaking the central symmetry observed under pure heat conduction. These results reveal that inclusion evolution is controlled by a delicate balance among reaction, diffusion, and interface effects.

The present framework successfully captures the simultaneous precipitation and co-evolution of Al_2_O_3_ and SiO_2_ inclusions. Increasing the initial oxygen concentration reduces the growth asymmetry arising from differences in thermodynamic driving forces, leading to progressively more symmetric growth of the two phases under oxygen-rich conditions. Overall, the morphological evolution of composite inclusions is closely associated with the coupled reaction, diffusion, and liquid–solid phase transformation processes, and is essentially controlled by the balance between oxide-solute supply and transformation kinetics during competitive growth.

The formation of dendritic inclusions is primarily attributed to anisotropy-induced interfacial instability, which promotes preferential growth along specific directions. The anisotropy strength and symmetry order determine the morphological symmetry and growth orientation of dendrites, while the development of branching is governed by the interplay between the availability of dissolved Al_2_O_3_ species and interface kinetics. In addition, the gradient energy coefficient regulates the interfacial stability and characteristic length scale, controlling the transition between compact and dendritic morphologies.

Future work will focus on quantitative validation against selected steel grades using experimentally calibrated parameters to determine whether the same qualitative trends hold under realistic material properties. In addition, three-dimensional extension of the model and the influence of dynamic thermal boundary conditions on inclusion precipitation will also be explored. Future work may also extend the model to include fluid flow, electromagnetic stirring, inclusion transport, and temperature-dependent material properties, which would further extend its applicability to practical steelmaking processes. Nevertheless, the present framework provides a useful basis for investigating reactive precipitation and morphological evolution of oxide inclusions in molten steel.

## Figures and Tables

**Table 1:** List of main dimensionless parameters.

Parameters	Definition	Parameters	Definition	Parameters	Definition
${\bar{L}}_{c}$	$\frac{L_{c}RT_{0}t_{0}}{c_{0}}$	${\bar{\mu }}^{\circ }$	$\frac{\mu^{\circ }}{RT_{0}}$	${\bar{C}}_{R}$	$\frac{C_{R}}{R}$
${\bar{f}}^{a}$	$\frac{f^{a}}{RT_{0}c_{0}}$	$\bar{D}$	$\frac{Dt_{0}}{l_{0}^{2}}$	${\bar{\rho }}^{M}$	$\frac{\rho^{M}}{c_{0}}$
${\bar{L}}_{\varphi }$	$L_{\varphi }RT_{0}c_{0}t_{0}$	${\bar{\kappa }}^{\varphi }$	$\frac{\kappa^{\varphi }}{{{RT_{0}c_{0}l}_{0}}^{2}}$	${\bar{f}}^{P}$	$\frac{f^{P}}{RT_{0}c_{0}}$
${\bar{\kappa }}^{T}$	$\frac{\kappa^{T}t_{0}}{Rc_{0}l_{0}^{2}}$	$∆{\bar{H}}_{r}$	$\frac{∆H_{r}}{RT_{0}}$	$∆{\bar{H}}_{sol}$	$\frac{∆H_{sol}}{RT_{0}}$

**Table 2:** Reference values of model parameters.

Parameters	Values	Parameters	Values
$c_{0}$	0.2 $mol/{cm}^{3}$	$T_{0}$	1873 K [38]
${\bar{c}}_{initial}$([Al])	1	${\bar{c}}_{initial}$([O])	0.5
${\bar{c}}_{initial}$($M^{A}$)	0	${\bar{c}}_{initial}$(Al_2_O_3_(L))	0
${\bar{T}}_{initial}$	1	$\varphi_{max,initial}$	0.99
${\bar{L}}_{c}$	2	$∆G^{{}^{\circ}}$	−612,500+196.9T J/mol [38]
${\bar{C}}_{R}$	1	γ	0.02
$f^{a}$	10,000 $J/{cm}^{3}$	κ	1
$\bar{D}$	0.002	M(Al_2_O_3_(s))	102 g/mol
${\bar{L}}_{\varphi }$	1	${\bar{\kappa }}_{\circ }^{\varphi }$	0.015
ϵ	0	m	0
ρ(Al_2_O_3_(s))	3.97 $g/{cm}^{3}$	$c_{T}$(Al_2_O_3_(s))	1 J/g·K
$\rho_{l}$	7.04 $g/{cm}^{3}$	${c_{T}}_{l}$	0.8 J/g·K
${\bar{\kappa }}^{T}$(Al_2_O_3_(s))	0.001	${\bar{\kappa }}_{l}^{T}$	0.002
$∆H_{r}$	$\left[\begin{array}{c} 10,000\\ -522,500 \end{array}\right]\;J/mol$	$∆H_{sol}$	100,000 J/mol

**Table 3:** Time-step sensitivity test based on the mean ${\bar{c}}_{\left(Al2O3\left(L\right)\right)}$ and mean φ.

$∆\bar{t}$	Mean $\bar{c}$_(Al2O3(L))_	Mean φ
0.00005	0.144024	0.03694
0.0001	0.144039	0.03687
0.0002	0.144055	0.03679

**Table 4:** Grid-independence test based on the mean ${\bar{c}}_{\left(Al2O3\left(L\right)\right)}$ and mean φ.

Grid	Mean $\bar{c}$_(Al2O3(L))_	Relative Change	Mean φ	Relative Change
256 × 256	0.13944	-	0.07898	-
512 × 512	0.13655	2.07%	0.09513	20.45%
1024 × 1024	0.13558	0.71%	0.10074	5.90%
2048 × 2048	0.13527	0.23%	0.10252	1.77%

## Data Availability

The original contributions presented in this study are included in the article. Further inquiries can be directed to the corresponding author.

## References

[1] Zhang L.F., Thomas B.G. (2003). State of the art in evaluation and control of steel cleanliness. ISIJ Int..

[2] Zhang L., Thomas B.G., Wang X., Cai K. Evaluation and control of steel cleanliness-review. Proceedings of the Steelmaking Conference Proceedings.

[3] Wang Y., Karasev A., Park J.H., Jönsson P.G. (2021). Non-metallic inclusions in different ferroalloys and their effect on the steel quality: A review. Metall. Mater. Trans. B-Proc. Metall. Mater. Proc. Sci..

[4] Murakami Y., Kodama S., Konuma S. (1989). Quantitative-evaluation of effects of non-metallic inclusions on fatigue-strength of high-strength steels. I: Basic fatigue mechanism and evaluation of correlation between the fatigue fracture-stress and the size and location of non-metallic inclusions. Int. J. Fatigue.

[5] Silva A. (2019). The effects of non-metallic inclusions on properties relevant to the performance of steel in structural and mechanical applications. J. Mater. Res. Technol.-JMRT.

[6] Vantadori S., Ronchei C., Scorza D., Zanichelli A., Araújo L.C., Araújo J.A. (2022). Influence of non-metallic inclusions on the high cycle fatigue strength of steels. Int. J. Fatigue.

[7] Zhao Y.X., Ren G.Q., Chen L.M., Gu G.Q., Zhu J.C., Zhao A.G. (2024). Influence of non-metallic inclusions on very high-cycle fatigue performance of high-strength steels and interpretation via crystal plasticity finite element method. Metals.

[8] Liu C., Gao X., Ueda S., Guo M., Kitamura S.-Y. (2020). Composition changes of inclusions by reaction with slag and refractory: A review. ISIJ Int..

[9] Wang Z., Bao Y. (2024). Development and prospects of molten steel deoxidation in steelmaking process. Int. J. Miner. Metall. Mater..

[10] Ferreira I.L. (2024). Assessment of thermodynamic variables affecting phase nucleation. Phys. B.

[11] Gao D.X., Tang X.Y., Wang X., Yang X., Zhang P.J., Che G.W., Han J., Hattori T., Wang Y.J., Dong X. (2021). Phase transition and chemical reactivity of 1H-tetrazole under high pressure up to 100 GPa. Phys. Chem. Chem. Phys..

[12] Yang Q.Q., Yuan C.S., Liang Y.F., Zhu X., Wang Z., Cheng X.R., Su L. (2025). Phase transition and chemical stability of s-Triazine under static and dynamic compression. Chem. Phys. Lett..

[13] He F., Xie S.P., He C.Y., Zhao Y.T. (2025). Phase change transpiration cooling of propylene glycol solution considering pyrolysis. Phys. Fluids.

[14] Ling Z.B., Diao F.Y., Sheng X.L., Li T., Cai R.S., Wang Y.Q. (2023). Chemical reaction and phase transformation mechanism of electrospun iron (III) acetylacetonate-polyacrylonitrile fibers during pre-oxidation process. Chem. Phys. Lett..

[15] Enomoto T., Akimoto A.M., Yoshida R. (2025). Chemically-fueled phase transition of a redox-responsive polymer. Sci. Technol. Adv. Mater..

[16] Donau C., Spath F., Stasi M., Bergmann A.M., Boekhoven J. (2022). Phase transitions in chemically fueled, multiphase complex coacervate droplets. Angew. Chem. Int. Ed..

[17] Cheng Z.M., Anter A.M., Yuan W.K. (2001). Intensification of phase transition on multiphase reactions. AIChE J..

[18] Saher S., Johnston S., Esther-Kelvin R., Pringle J.M., MacFarlane D.R., Matuszek K. (2024). Trimodal thermal energy storage material for renewable energy applications. Nature.

[19] Schlogl F. (1972). Chemical reaction models for non-equilibrium phase transitions. Z. Fur Phys..

[20] Nguyen B., Seifert U. (2020). Exponential volume dependence of entropy-current fluctuations at first-order phase transitions in chemical reaction networks. Phys. Rev. E.

[21] Dakhnovskii Y.I., Ovchinnikov A.A., Benderskii V.A. (1982). Enhancement of low-temperature chemical-reactions in phase-transitions. Chem. Phys..

[22] Bothe D., Prüss J. (2017). Modeling and analysis of reactive multi-component two-phase flows with mass transfer and phase transition —The isothermal incompressible case. Discret. Contin. Dyn. Syst. Ser. S.

[23] Dreyer W., Giesselmann J., Kraus C. (2014). A compressible mixture model with phase transition. Phys. D.

[24] Wall M.A., Cossairt B.M., Liu J.T.C. (2018). Reaction-driven nucleation theory. J. Phys. Chem. C.

[25] Zhang Y.X., Liang H.M., Zhang Q. (2024). Coupling mechanism of physical processes and chemical reactions during phase transition in liquid tanks under thermal radiation. Process Saf. Environ. Prot..

[26] Williams C.W., Srivastava G., Matous K. (2023). Continuum modeling predictions of nonlinear specific heat in phase transition of energetic materials. J. Mech. Phys. Solids.

[27] Li S.J., Liu J., Huang W.Y., Zhang C.H. (2024). Numerical simulation of the thermo-hydro-chemical coupling in enhanced geothermal systems: Impact of SiO_2_ dissolution/precipitation in matrix and fractures. Energy.

[28] Chen Y., Ma G.W., Wang H.D. (2018). The simulation of thermo-hydro-chemical coupled heat extraction process in fractured geothermal reservoir. Appl. Therm. Eng..

[29] Chen L.Q. (2002). Phase-field models for microstructure evolution. Ann. Rev. Mater. Res..

[30] Moelans N., Blanpain B., Wollants P. (2008). An introduction to phase-field modeling of microstructure evolution. Calphad-Comput. Coupling Phase Diagr. Thermochem..

[31] Xu Z.J., Meakin P. (2011). Phase-field modeling of two-dimensional solute precipitation/dissolution: Solid fingers and diffusion-limited precipitation. J. Chem. Phys..

[32] Xu Z.J., Huang H., Li X.Y., Meakin P. (2012). Phase field and level set methods for modeling solute precipitation and/or dissolution. Comput. Phys. Commun..

[33] Fang X.R., Liu Y.J., Xia Y. (2025). A coupled chemo-mechanical phase field model of sulfate induced cracking in concrete with porosity evolution. Eng. Fract. Mech..

[34] Zhu L.B., Liu W., Yang S.F., Li J.S., Wang F., Zhang X.L., Minerals M., Mat S. Numerical simulation of three-phase flow of gas-stirring micro-phenomenon during ladle furnace process. Proceedings of the 148th The-Minerals-Metals-and-Materials-Society (TMS) Annual Meeting and Exhibition (TMS) on Microelectronic Packaging, Interconnect, and Pb-Free Solder.

[35] Xuan C.J., Persson E.S., Jensen J., Sevastopolev R., Nzotta M. (2020). A novel evolution mechanism of Mg-Al-oxides in liquid steel: Integration of chemical reaction and coalescence-collision. J. Alloy. Compd..

[36] Liu W., Liu J., Zhao H.X., Yang S.F., Li J.S. (2021). CFD modeling of solid inclusion motion and separation from liquid steel to molten slag. Metall. Mater. Trans. B—Proc. Metall. Mater. Proc. Sci..

[37] Cao Z., Zhao P. (2026). A phase field model for reactive liquid-solid systems. ChemRxiv.

[38] Park J.H., Todoroki H. (2010). Control of MgO•Al_2_O_3_ spinel inclusions in stainless steels. ISIJ Int..

[39] Wu Z.H., Zheng W., Li G.Q., Matsuura H., Tsukihashi F. (2015). Effect of inclusions’ behavior on the microstructure in Al-Ti deoxidized and magnesium-treated steel with different aluminum contents. Metall. Mater. Trans. B-Proc. Metall. Mater. Proc. Sci..

[40] Liu C., Yuan H., Li X.D., Che Z.C., Yang S.F., Du C.W. (2022). Initiation mechanism of localized corrosion induced by Al_2_O_3_-MnS composite inclusion in low-alloy structural steel. Metals.

[41] Van Ende M.A., Guo M., Proost J., Blanpain B., Wollants P. (2010). Morphology of Al_2_O_3_ inclusions formed at Fe/Fe-Al interface. Ironmak. Steelmak..

[42] Fuseini N.I., O’Malley R.J., Sander T.P., Smith J.D., Wen H.M., Bartlett L.N. (2026). Post-calcium treatment evolution of alumina (Al_2_O_3_) inclusions morphology. Metall. Mater. Trans. B-Proc. Metall. Mater. Proc. Sci..

